# Aquaporin 4-positive neuromyelitis optica spectrum disorder with meningoencephalitis-like onset: A case report

**DOI:** 10.3389/fimmu.2022.938492

**Published:** 2022-10-06

**Authors:** Yi Bu, Heng Liu, XuDong Qian, Fan Sun, ChengBo Li, Jingzhe Han

**Affiliations:** ^1^ Department of Neurology, affiliated Hospital of Chengde Medical University, Chengde, China; ^2^ Department of Pain, Hengshui People’s Hospital, Hengshui, China; ^3^ Department of Neurology, Hengshui People’s Hospital, Hengshui, China

**Keywords:** neuromyelitis optica spectrum disease, aquaporin, area postrema syndrome, central nervous system, autoimmune inflammatory demyelinating disease

## Abstract

Neuromyelitis optica spectrum disease (NMOSD) is a debilitating autoimmune inflammatory demyelinating disease of the central nervous system. The relationship between harboring an infection and NMOSD is currently unclear and needs further investigation. This article reports meningoencephalitis-like manifestations, including fever, headache, neck resistance, seizures, and pleocytosis, accompanied by nausea and vomiting, in a patient with serum AQP4 antibody-positive area postrema syndrome (APS). In the presence of aseptic meningitis combined with clinical symptoms such as optic neuritis and myelitis, the possibility of NMOSD diagnosis can be considered. However, for patients with unknown causes, especially combined with aseptic meningitis, a probable differential diagnosis of NMOSD is considered.

## Introduction

Neuromyelitis optica spectrum disease (NMOSD) is a debilitating autoimmune inflammatory demyelinating disease of the central nervous system. The condition is associated with optic neuritis, acute myelitis, terminal medulla syndrome and acute brainstem syndrome. Other features include acute diencephalic syndrome and acute cerebral syndrome. Aquaporin 4 (AQP4) antibody is a biomarker closely related to the pathogenesis of NMOSD (1). Patients with a previous diagnosis of intracranial infection ends up harboring antibody-related meningoencephalitis or meningitis, such as autoimmune glial fibrillary acidic protein astrocyte Disease (A-GFAP-A) and MOG antibody-related diseases. The observed relationship between infection and immunity needs further investigation. Sporadic meningitis or meningoencephalitis cases present as the primary clinical manifestation of AQP4 antibody-positive NMOSD.

For most patients, aseptic meningitis might be suspected early or later diagnosed as suffering from meningitis-like manifestations of NMOSD (2–6). However, these patients were almost accompanied by the clinical and imaging features of central nervous system damage, such as myelitis and optic neuritis, at the same time when they presented with the symptoms of aseptic meningoencephalitis.

This article reports a case of NMOSD with aseptic meningoencephalitis (fever, headache, neck resistance, seizures, and pleocytosis) as the primary manifestation, accompanying INH (intractable nausea, vomiting, and hiccups), but lacks typical imaging features of APS, which is very rare and easily misdiagnosed. Since it would be difficult to suspect that the patient could have APS, especially in the absence of typical imaging features. Our hospital ethics committee approved this case report. We obtained the patient’s informed consent for treatment and de-identified all patient details. The reporting of this study conforms to CARE guidelines (7).

## Case illustration

**Figure 1:**
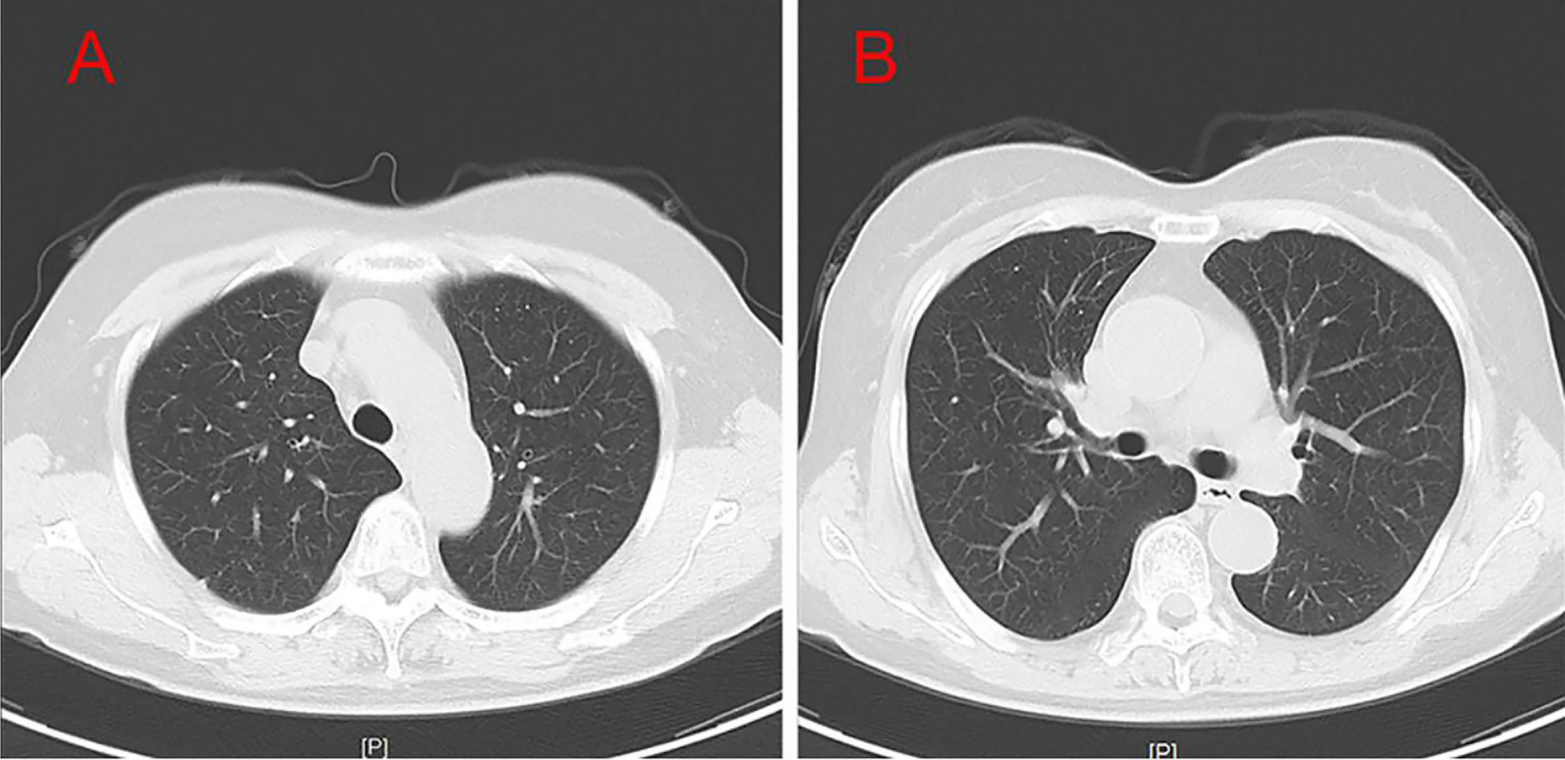
Test results of CT **(A, B)**. Chest CT results showed that the patient had bronchitis and multiple lung nodules.

**Figure 2:**
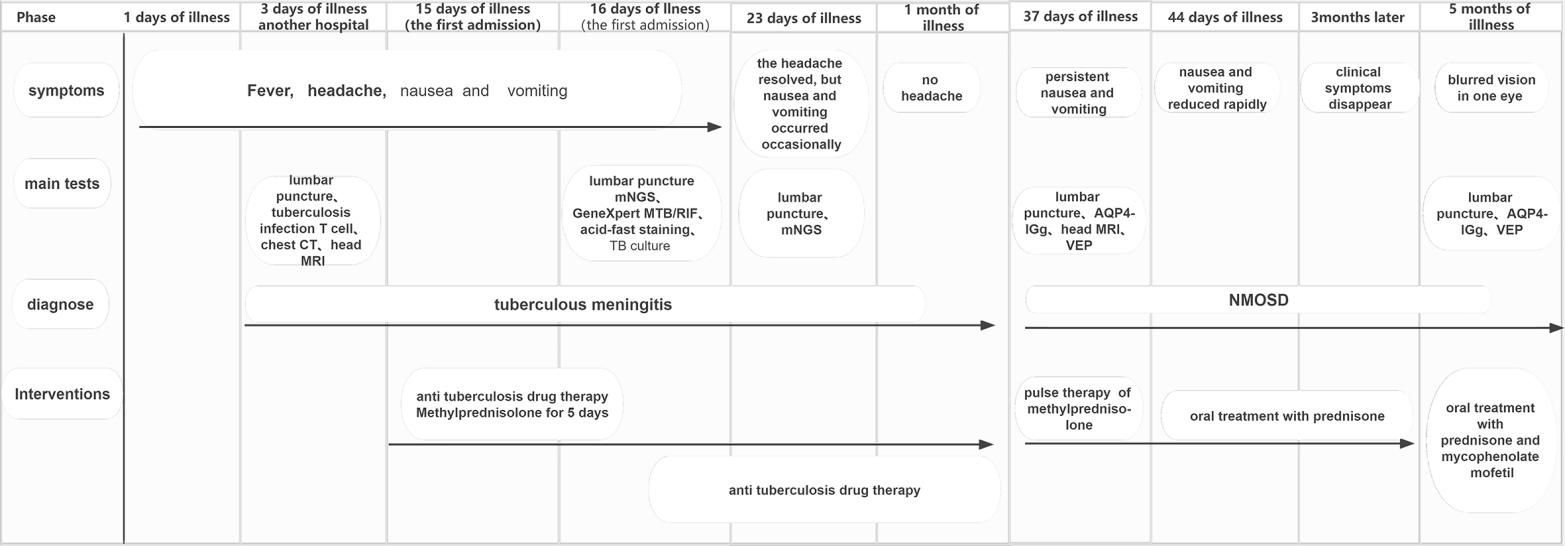
Symptoms and interventions of patients before and after treatment.

**Figure 3:**
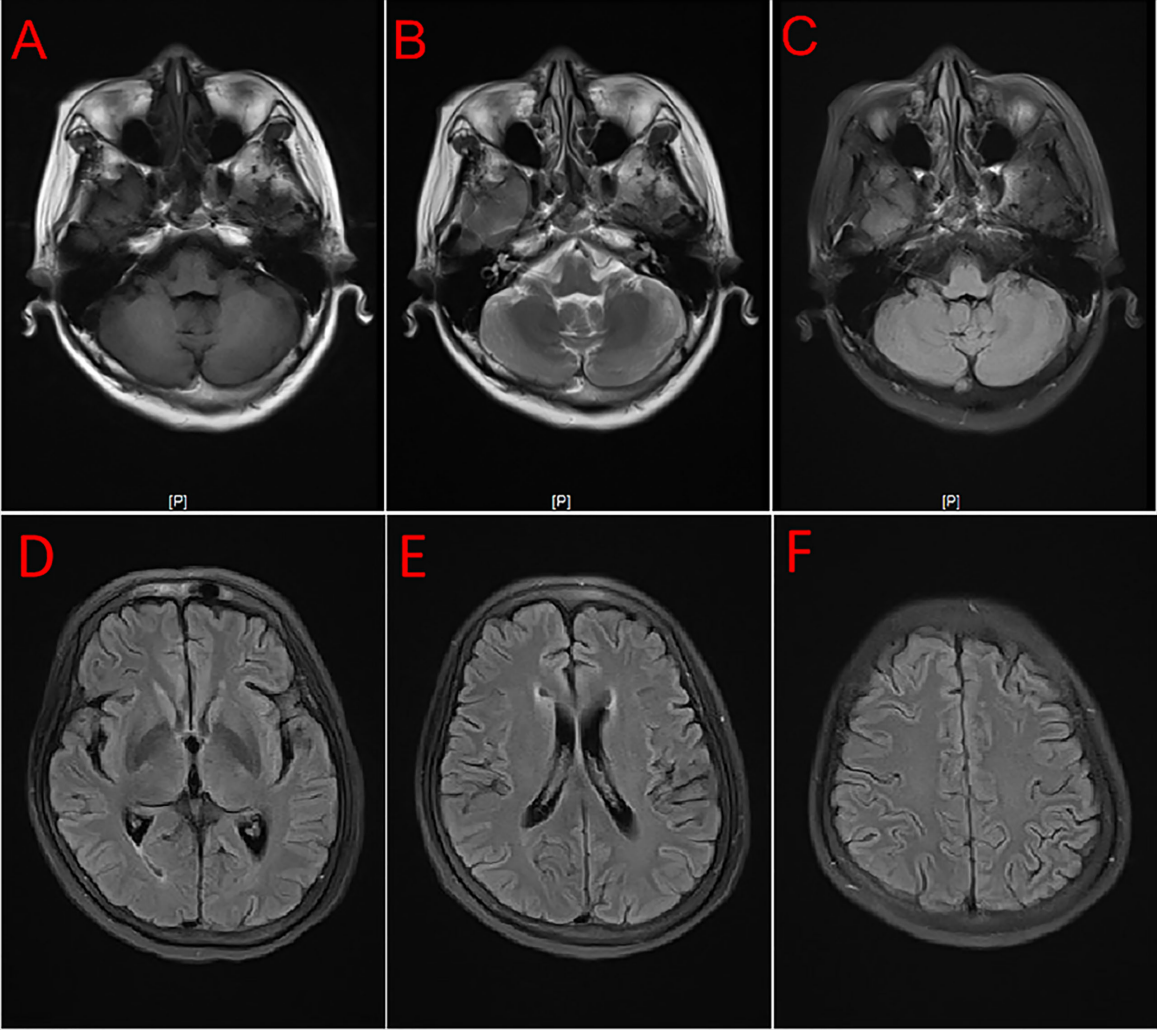
MRI showed no typical imaging features for APS and brain lesions. T1 **(A)**, T2 **(B)**, and Flair **(C–F)**.

A 54-year-old female patient was admitted to the hospital for 14 days, complaining of fever, headache, nausea, and vomiting. The temperature at the time of admission was 38.5°C. History taking revealed that the patient had undergone a lumbar puncture some days ago in a hospital. The lumbar pressure was 120 mmH_2_O, white blood cell count was 281×106/L, the cerebrospinal fluid protein was 1139 mg/L, cerebrospinal fluid glucose 2.50 mmol/L, and cerebrospinal fluid chloride was 117.8 mmol/L (**Table 1**). The lady tested positive for serum tuberculosis infection T cell, and a diagnosis of tuberculous meningitis was made. The patient experienced intermittent limb twitching daily to the present admission, accompanied by loss of consciousness. In addition, the patient confessed to having a history of “diabetes” for about half a month. Physical examination was conducted upon admission. The patient had a positive result for the neck resistance test, and the head MRI showed lacunar infarction in the left basal ganglia. A Chest CT scan revealed multiple nodules in both lungs (**Figure 1**). Organ function tests relating to the liver and kidney returned typical results. The sodium and chloride ion levels were 124.4mmol/L and 92.2mmol/L, respectively. Both HIV antibody and syphilis monitoring tests were negative. A diagnosis of tuberculous meningitis was made, and the patient was given antituberculosis drug therapy comprising isoniazid, rifampicin, and pyrazinamide. Re-examining the lumbar puncture on the second day of admission showed a lumbar pressure of 150 mmH_2_O, white blood cell count was 44×106/L, the cerebrospinal fluid protein was 636 mg/L, cerebrospinal fluid glucose was 5.84 mmol/L, and cerebrospinal fluid chloride was 111.8 mmol/L (**Table 1**). Cerebrospinal fluid cytology was suggestive of mixed cell response. No abnormalities were seen regarding cryptococcal capsular antigen and acid-fast staining tests, cerebrospinal fluid mNGS (metagenomics next generation sequencing), culture and Gene Xpert MTB/RIF. Similarly, there were no pathogenic microorganisms found in the cerebrospinal fluid. The EEG results showed slowed brain wave frequencies during the awake period, and moderately slow waves (3-5 Hz) appeared intermittently in each lead. After instituting anti-tuberculosis treatment, the clinical symptoms improved rapidly; the headache resolved, but nausea and vomiting occasionally occurred (**Figure 2**). One week after admission, the lumbar puncture was repeated, giving the following results: lumbar pressure was 75 mmH_2_O, white blood cell count was 42×10^6^/L, cerebrospinal fluid protein was 651 mg/L, cerebrospinal fluid glucose was 6.30 mmol/L, and cerebrospinal fluid chloride was 110.9 mmol/L (**Table 1**). Re-examination of the cerebrospinal fluid mNGS found no pathogenic microorganisms. Two weeks after admission, the patient had no headache and was discharged from the hospital on oral antituberculosis drugs. However, one week after being discharged from the hospital, the patient was re-admitted to the hospital, complaining of persistent nausea and vomiting. A re-examination of the lumbar puncture showed that the pressure was 100 mmH_2_O, the number of white blood cells was 23×10^6^/L, cerebrospinal fluid protein 504 mg/L, and cerebrospinal fluid chloride was 108.1mmol/L. Blood sodium and chloride ions was 128.5 mmol/L and 90.9 mmol/L, respectively (**Table 1**). A re-examination of the head MRI showed no typical APS imaging features (**Figure 3**). The serum AQP4 antibody test (Cytometric Bead Array method) was 1:100, while the fundus examination, visual examination, evoked potential, and spinal cord magnetic resonance was negative. Besides, anti-MOG, anti-GFAP, anti-NMDA, anti-IgG4 antibodies, and autoantibodies were all negative, leading to a definite diagnosis of NMOSD. At the same time, antituberculosis drugs were discontinued. After instituting pulse therapy using 1000 mg of methylprednisolone, nausea and vomiting reduced rapidly. Six days later, the patient was discharged on oral treatment with prednisone. Three months later, the patient’s clinical symptoms did not recur. In addition, five months later, the patient developed blurred vision in one eye during the follow-up. VEP showed prolonged P100 latency and decreased P100 amplitude. The visual field test indicated a superior and temporal visual field defect, AQP4-Ab testing was reviewed, which titer was 1:10. Lumbar pressure 90 mmH_2_O, white blood cell count was 1×10^7^/L, cerebrospinal fluid protein was 312 mg/L, cerebrospinal fluid glucose 6.10 mmol/L, and cerebrospinal fluid chloride was 124.7mmol/L. Therefore, the possibility of optic neuritis was clinically considered, and mycophenolate mofetil was given. The patient’s vision recovered rapidly in a short time after the treatment.

**Table 1:** CSF features of patients before and after treatment.

Phase	CSF Pressure (mmH2O)	CSF WBC (10^6^/L) (lymphocyte%)	CSF Protein (mg/L)	CSF Glucose (mmol/L)	CSF Chloride (mmol/L)
3 days of illness (another hospital)	120	281(NA)	1139	2.50	117.8
16 days of illness (the first admission)	150	44(100%)	636	5.84	111.8
23 days of illness (the first admission)	75	42(100%)	651	6.30	110.9
37days of illness (the second admission)	100	23(100%)	504	6.70	108.1
5 months of illness (follow up)	90	10(100%)	312	6.10	124.7

## Discussion

The relationship between harboring an infection and NMOSD is currently unclear, although infections are considered one of the causes of autoimmune diseases. For example, herpes zoster virus infection has been linked to NMOSD (8, 9). In their work, Koga and colleagues tested 19 patients with AQP4 antibody-positive NMOSD for viral and bacterial infections. The researchers concluded that a previous infection might be a trigger for NMOSD. However, all the patients in the above study did not have any meningoencephalitis-like clinical features such as fever, headache, and neck stiffness (10). The patient presented with fever, headache, neck resistance, and epilepsy as the initial clinical manifestations in the current case report. Results based on a combination of clinical manifestations and laboratory examinations were suggestive of tuberculous meningitis. The final diagnosis of NMOSD was an unexpected, unique feature of this case. The early tuberculous meningitis-like clinical features the patient presented are pretty baffling. Meningoencephalitis-like symptoms such as fever and headache can be symptomatic of an NMOSD episode or previous infection or trigger of NMOSD. Therefore, the differential diagnosis of this patient should mainly exclude infectious diseases, including tuberculous meningitis. However, in our comprehensive analysis of the patient, the diagnosis of TB meningitis at first was not reliable for the following reasons: there was no etiological evidence of tuberculosis infection throughout the disease; symptoms of fever and headache improved rapidly within days of anti-tuberculosis and glucocorticoid therapy (this was not consistent with the outcome of tuberculous meningitis); after less than 1 month of anti-tuberculosis treatment, cerebrospinal fluid improved rapidly, which was almost impossible in patients with tuberculous meningitis. Since only a small number of patients with suspected viral meningitis or encephalitis can be clinically identified with a definite pathogen. Therefore, a diagnosis of viral meningitis in a patient cannot be completely ruled out. Although the early cerebrospinal fluid changes in this patient do not support viral infection, there is some risk of complete exclusion. Cerebrospinal fluid pathogen mNGS has a high diagnostic value in intracranial pathogenic infections, and a negative result can rule out most pathogenic infections. However, the use of mNGS is limited by disadvantages such as low sensitivity when testing and susceptibility to false-negative results. Therefore, in this study, we concluded that the patient’s early symptoms might be due to aseptic meningitis caused by NMOSD attacking the central nervous system. However, other pathogenic conditions still cannot be excluded entirely.

As an exceptionally rare phenotype of NMOSD, aseptic meningitis has been reported in only a few cases of AQP4 antibody-seropositive NMOSD, in which most patients initially presented with aseptic meningitis. In contrast, others experienced meningitis several years after diagnosing NMOSD (2–6). Although rare, pathological and clinical studies have confirmed pia mater involvement in NMOSD including headache, meningeal irritation and leptomeningeal enhancement on MRI (6). Direct meningeal involvement may be associated with the development of meningoencephalitis-like symptoms in this patient. The occurrence of this symptom may also be related to the occurrence of hypersensitivity reactions in the central nervous system. The exact mechanism of meningoencephalitis-like symptoms needs to be further investigated. According to scientific reports, about 13-35% of patients with NMOSD have a cerebrospinal fluid white cell count greater than 50×10^6^/L (11). Immune diseases involving the central nervous system can cause a slight decrease in the cerebrospinal fluid sugar content and chloride levels. Therefore, the clinical manifestations and the results of this patient’s cerebral spinal fluid analysis reflected those experienced with tuberculous meningitis, making it difficult to make a differential diagnosis. The significant increase of white blood cells and proteins of CSF (cerebrospinal fluid) in our patient may be related to the damage of BBB (blood-brain barrier) and aseptic inflammatory response. The decrease of CSF chloride may be related to CSF protein or serum chloride. The possible mechanisms of low CSF glucose are as follows: the increased glucose exudation of CSF is caused by the damage of BBB; local cerebrovascular inflammation leads to vasoconstriction or stenosis, which results in abnormal cerebral hemodynamics and reducing glucose transport into CSF.

Intractable nausea or vomiting and hiccups that other reasons cannot explain are referred to as APS and are among the most characteristic clinical symptoms of NMOSD. The condition can suggest NMOSD before spinal cord or optic nerve damage (12). Although the typical imaging characteristics of APS were lacking in our patient, characterized by frequent unexplained nausea and vomiting, the diagnosis was finally confirmed by a positive serum AQP4-antibody. A reported that the sensitivity and specificity of the CBA method are 91% and 100% (13). Furthermore, nausea and vomiting disappeared after therapy, further supporting the diagnosis of APS. Meningoencephalitis is often associated with nausea and vomiting, making it challenging to distinguish APS from symptoms of meningoencephalitis. We also experienced this challenge with the current case study leading to a delayed definitive diagnosis of APS. More importantly, and as mentioned earlier, this patient lacked the imaging features of APS, which further complicated reaching an early diagnosis of the condition. It has been suggested that when a patient has INH and imaging does not reveal pathological features corresponding to APS, NMOSD cannot be ruled out (14). Therefore, more emphasis should be placed on clinical features rather than imaging evidence when pursuing a diagnosis of APS.

The most common adverse effect of anti-tuberculosis drugs is gastrointestinal symptoms. This patient’s intractable nausea and vomiting are likely related to the administration of anti-tuberculosis drugs. Given this factor, the symptoms above were not taken seriously at first. The above is one of the main reasons for the delayed diagnosis of the patient. The dosage of anti-tuberculosis drugs was adjusted constantly during the first admission. However, no significant improvement was found in nausea and vomiting. After the first discharge, the patient voluntarily stopped the anti-tuberculosis drugs due to nausea and vomiting, but nothing worked, and the symptoms continued. The patient’s vomiting symptoms showed no significant improvement at the second admission. The response of this patient’s nausea and vomiting symptoms to hormones suggests that the onset of symptoms may be related to immune factors. Many autoimmune diseases can lead to meningoencephalitis-like symptoms. Patients often present with nausea, vomiting, and elevated cerebrospinal fluid leukocytes. The possibility of autoimmune disease was excluded based on serological, cerebrospinal fluid antibodies, and imaging tests. The presence of infections or other immune-related diseases must be further confirmed by follow-up.

## Conclusions

NMOSD may be easily diagnosed if a patient presents with aseptic meningitis combined with clinical signs such as optic neuritis and myelitis. However, for patients with frequent nausea and vomiting of unknown causes, especially combined with aseptic meningitis, a probable differential diagnosis of NMOSD should be considered. It is essential to eliminate interfering factors and identify the APS to accurately avoid a misdiagnosis of NMOSD. In addition, cerebrospinal fluid mNGS technology can have some diagnostic value in providing a relationship between infection and immunity, especially for NMOSD patients with aseptic meningitis.

## Data availability statement

The original contributions presented in the study are included in the article/supplementary material. Further inquiries can be directed to the corresponding author.

## Ethics statement

The studies involving human participants were reviewed and approved by the Ethics Committee of Hengshui People’s Peace Hospital. The patients/participants provided their written informed consent to participate in this study. Written informed consent was obtained from the individual(s) for the publication of any potentially identifiable images or data included in this article.

## Author contributions

(1)Design or conceptualization of the study. (2) Acquisition of the data. (3) Analysis or interpretation of the data. (4) Drafting the initial draft.(5) Revising the manuscript. (6)Supervision. (7): Funding acquisition. YB and HL has contributed to 1, 2 and 5. XQ and FS has contributed to 2,3. CL has contributed to 4. JH has contributed to 6,7. All authors contributed to the article and approved the submitted version.

## Funding

This study was funded by the Science and Technology Planning Project of Hengshui city in 2019 (2019014078Z), China.

## Conflict of interest

The authors declare that the research was conducted in the absence of any commercial or financial relationships that could be construed as a potential conflict of interest.

## Publisher’s note

All claims expressed in this article are solely those of the authors and do not necessarily represent those of their affiliated organizations, or those of the publisher, the editors and the reviewers. Any product that may be evaluated in this article, or claim that may be made by its manufacturer, is not guaranteed or endorsed by the publisher.
